# Transcriptome and Gene Regulatory Network Analyses Reveal New Transcription Factors in Mature Fruit Associated with Harvest Date in *Prunus persica*

**DOI:** 10.3390/plants11243473

**Published:** 2022-12-12

**Authors:** Gerardo Núñez-Lillo, Wellasmin Pérez-Reyes, Anibal Riveros, Victoria Lillo-Carmona, Karin Rothkegel, José Miguel Álvarez, Francisca Blanco-Herrera, Romina Pedreschi, Reinaldo Campos-Vargas, Claudio Meneses

**Affiliations:** 1 Escuela de Agronomía, Facultad de Ciencias Agronómicas y de los Alimentos, Pontificia Universidad Católica de Valparaíso, Quillota 2260000, Chile; 2 Centro de Biotecnología Vegetal, Facultad de Ciencias de la Vida, Universidad Andrés Bello, Santiago 8370186, Chile; 3 Departamento de Fruticultura y Enología, Facultad de Agronomía e Ingeniería Forestal, Pontificia Universidad Católica de Chile, Santiago 7820436, Chile; 4 Departamento de Genética Molecular y Microbiología, Facultad de Ciencias Biológicas, Pontificia Universidad Católica de Chile, Santiago 8331150, Chile; 5 ANID-Millennium Science Initiative Program, Millennium Nucleus for the Development of Super Adaptable Plants (MN-SAP), Santiago 8331150, Chile; 6 Millennium Institute Center for Genome Regulation (CRG), Santiago 8331150, Chile; 7 Departamento de Producción Agrícola, Facultad de Ciencias Agronómicas, Universidad de Chile, Santiago 8820808, Chile

**Keywords:** fruit ripening, transcriptomics, network analysis, transcription factors, peach development

## Abstract

Harvest date is a critical parameter for producers and consumers regarding agro-industrial performance. It involves a pleiotropic effect controlling the development of other fruit quality traits through finely controlling regulatory mechanisms. Fruit ripening is a process in which various signals and biological events co-occur and are regulated by hormone signaling that produces the accumulation/degradation of multiple compounds. However, the regulatory mechanisms that control the hormone signaling involved in fruit development and ripening are still unclear. To investigate the issue, we used individuals with early, middle and late harvest dates from a peach segregating population to identify regulatory candidate genes controlling fruit quality traits at the harvest stage and validate them in contrasting peach varieties for this trait. We identified 467 and 654 differentially expressed genes for early and late harvest through a transcriptomic approach. In addition, using the Arabidopsis DAP-seq database and network analysis, six transcription factors were selected. Our results suggest significant hormonal balance and cell wall composition/structure differences between early and late harvest samples. Thus, we propose that higher expression levels of the transcription factors *HB7*, *ERF017* and *WRKY70* in early harvest individuals would induce the expression of genes associated with the jasmonic acid pathway, photosynthesis and gibberellins inhibition. While on the other hand, the high expression levels of *LHY*, *CDF3* and *NAC083* in late harvest individuals would promote the induction of genes associated with abscisic acid biosynthesis, auxins and cell wall remodeling.

## 1. Introduction

Peaches and nectarines (*Prunus persica* (L.) Batsch) are one of the most important temperate climate fruit crops in the world based on fruit production (24,665,205 tons; FAOSTAT, 2019) and have been used as model systems for genetic and genomic studies within its genus [1]. In Chile, peach fruit production starts in November and ends in April, where early, middle and late harvest varieties can be identified with marked differences in development, fruit quality and postharvest life [2]. Climacteric fruit, such as peaches and nectarines, are characterized by increased ethylene production in the last stage of the ripening process, causing fruit deterioration and affecting shelf life [3]. The Chilean peach export industry relies on good quality products for markets such as Europe, the US and Asia, with this being a challenge considering the 30 to 45 days of transport under low-temperature conditions [4]. Consequently, losses of up to 15% of the total exported production are generated. For these reasons, peach breeding programs have focused for 30 years on developing new varieties with improved fruit quality traits and postharvest performance [5].

Harvest date is a critical parameter for producers in agro-industrial performance since selecting cultivars with contrasting harvest dates would be advantageous to cover and extend the marketing season [6]. From a consumer point of view, this trait not only determines the optimal harvest date in terms of organoleptic quality, but also has a pleiotropic effect, controlling the development of other fruit quality traits such as softening, sugar accumulation and acidity [6,7,8,9]. Previous studies on early, middle and late harvest varieties have shown that, in general, late harvest varieties display larger fruit sizes, are firmer, accumulate more sugars and have lower acidity [10]. For instance, Monti et al. [2] identified differences in the metabolic profiles of peach varieties with different harvest dates, such as a higher content of amino acids in early varieties and a higher content of maltose in varieties of middle and late harvests. In addition, Colantuono et al. [11] evaluated quality attributes in 26 peach varieties displaying different harvest dates, identifying correlations between acidity and susceptibility to mechanical damage or browning with early, middle and late harvest varieties. Thus, the harvest date phenotype should bring together many traits that warrant optimal organoleptic characteristics and postharvest performance.

Fruit ripening is a complex process that involves the interplay of various signals and biological events that take place at the same time. All of these processes have a fine-tuned hormonal regulation that affects cell signaling and produces the accumulation/degradation of various metabolites or alterations in cell wall composition. A candidate gene for harvest date was identified by Quantitative Trait Loci (QTL) analysis on chromosome 4 on the peach genome, described as transcription factor NAC072 [6,8,9]. In addition, two genomic variations were identified on this gene sequence that explain the observed variations in harvest date phenotype. The first is a nine bp deletion in the third gene exon [6], and the second is a 26.6 kbp deletion that removes the entire NAC072 gene from the peach genome [9]. This last homozygous deletion presents a phenotype that stops fruit development in the S2 stage (slow ripening) [12]. Transcriptomic analysis has revealed that the absence of this transcription factor generates differences in the expression of key genes involved in the abscisic acid (ABA) and gibberellic acid (GA) biosynthesis pathways in the early stages of fruit development [13].

Peaches are climacteric fruits and, as such, require autocatalytic ethylene for normal ripening [14]. The participation of ethylene as the main hormone that triggers ripening in climacteric fruits has been well described [15]. However, in recent years, the number of studies that show that other hormones have fundamental roles in different stages of fruit development has increased. For instance, the role of ABA in ripening has been associated mainly with non-climacteric fruits [16]. Recently, its role in climacteric fruit ripening has been related to the regulation of many developmental processes such as softening [17], anthocyanins and sugars accumulation [18] and even triggering ethylene biosynthesis [17,18]. On the other side, jasmonic acid (JA), a lipid-derived phytohormone that participates in defense responses, has been mainly associated with abiotic stress. However, its role in fruit ripening has also been described in recent years as slowing down peach fruit development through the downregulation of ethylene, cell wall and auxin-related genes, but increasing anthocyanin content [19,20,21,22]. On the contrary, in non-climacteric fruits such as *Fragaria chiloensis*, an inverse effect has been observed, where applications of methyl jasmonate accelerated fruit development [23].

A wide range of transcription factors associated with regulating plant hormonal levels has been described. Crosstalk between them makes hormonal regulation a very complex process. For example, transcription factors such as MYB101 and MYB33 have been reported to positively regulate ABA content in response to abiotic stress. In contrast, transcription factors such as ABI3 and ABI5 repress MYB101 and MYB33 during seed germination, thus controlling ABA levels [24]. Likewise, WRKY transcription factors (WRKY18, WRKY40 and WRKY60) were related to ABA in plant defense and abiotic stress response [25]. On the other hand, it has been widely reported that the JA signaling pathway is constantly repressed by JAZ proteins, which suppress the expression of various types of JA-response transcription factors such as MYC, MYB, WRKY, ERF and NAC. These transcription factors control plant processes such as development, defense or secondary metabolites biosynthesis. In particular, some WRKY and ERF transcription factors have been described as regulating JA biosynthesis [26]. Despite many reports on hormonal regulation, little is known about hormonal differences between fruit with different ripening speeds.

During ripening, fleshy fruits undergo changes in texture that lead to the loss of firmness and fruit softening. The softening process relies on different enzymes on specific cell wall sites, which hydrolyze components such as cellulose, hemicellulose, pectins and proteins [27,28]. Maturation and softening involve an increase in the activity of enzymes associated with cell wall depolymerization, such as exo- and endo-polygalacturonase, pectin methylesterase, endo-1,4-β-glucanase, α-arabinosidase and β-galactosidase. The intensity and duration of the increased enzymatic activity are independent for each enzyme, suggesting they are involved in different softening stages, making fruit softening a more complex process [28].

Previous studies have focused on understanding the complex regulation in fruit development and how this regulation affects fruit quality. However, few studies have focused on investigating the changes among individuals with different maturation speeds at the transcriptional regulation level. Thus, this work aimed to identify changes at the transcriptional level among early, middle and late harvest individuals from a peach-segregating population at the harvest stage. Specifically, candidate genes were selected and validated in peach varieties to construct a regulatory network that explains variations in fruit quality traits between peaches with different ripening speed.

## 2. Results

### 2.1. Fruit Quality Attributes Evaluation on Individuals with Contrasting Harvest Dates

For transcriptomic studies associated with the harvest date phenotype, a selection of nine individuals from a segregating population of ‘O×N’ peach trees with contrasting phenotypes was used (Table 1). The harvest date was determined according to its index of absorbance difference (I_AD_) described in the Materials and Methods section, between 0.8 and 1.2. The different phenotypic classes selected corresponded to 127.9, 136.8 and 153.3 days after bloom (DAB) for early, middle and late harvest individuals, respectively. In addition, as displayed in Table 1, these selected individuals were also segregated for other fruit quality traits such as weight (133.3 to 275.2 g), soluble solids content (8.8 to 14.4 Brix) and firmness (6.4 to 11.2 N).

On the other hand, two peach varieties with contrasting phenotypes for harvest date were selected and evaluated to validate the results observed in the ‘O×N’ segregating population. As shown in Table 1, the varieties “Big Boy” (BB) and “Late Red Jim” (LRJ) presented harvest dates at 104.5 and 170.0 DAB, respectively, being even more contrasting than those observed in the ‘O×N’ population. Furthermore, despite significant differences in fruit weight between the “Big Boy” (112.9 g) and “Late Red Jim” (207.6 g) varieties, no differences in soluble solids content or firmness were observed (15.6 Brix and 14.6 N on average, respectively).

### 2.2. RNAseq Analysis and Bioinformatic Results

**Figure 1:**
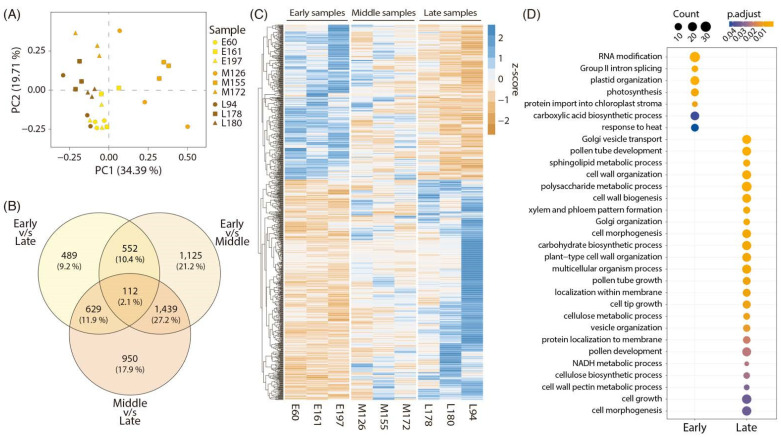
Differential expression analysis between contrasting harvest date samples from the ‘O×N’ population. (**A**) Principal component analysis using normalized counts of each sequenced library. Different colors represent different phenotypic classes. Early, middle and late harvest samples are represented with yellow, orange and brown colored symbols, respectively. The replicates of each selected individual are represented with different characters. (**B**) Venn diagram illustrating the differentially expressed transcripts of each comparison analyzed. (**C**) The candidate genes differentially expressed in the comparison of early vs. late with middle harvest expression values between them are represented in a blue–orange scale heatmap. Data were scaled using a z-score scaling method dividing the mean value of each gene by the standard deviation. Each column represents the average expression of three replicates for each individual selected. (**D**) Gene ontology term enrichment analyses genes overexpressed in early and late harvest samples. The blue–orange scale color represents the adjusted *p*-value, and the point size represents the gene ratio.

The sequencing results obtained from the 27 ‘O×N’ libraries selected (three harvest phenotypes with three biological replicates and three technical replicates) are shown in Table S1. On average, a total of 41,524,144 reads were obtained for each library, with minimum and maximum values of 29,675,066 and 53,190,150 reads, respectively. The read quality filters maintained 97.5% of total reads sequenced on average. After aligning them against the *Prunus persica* v2.0 reference genome [29], an average of 95.1% of total reads were correctly aligned. Normalized counts of each library were evaluated in a principal component analysis (PCA) to visualize the clustering of the biological replicates (Figure 1A). A clear separation of the different phenotypic classes studied (early, middle and late harvest date samples) was not observed; instead, a trend to group together was detected. In addition, the middle harvest samples displayed the most significant variance among their biological replicates. Differential expression analysis between the different phenotypic classes studied gave a total of 3228 differentially expressed genes (DEG) in the early vs. middle harvest comparison, 3130 DEGs in the middle vs. late harvest comparison and 1782 DEGs in the early vs. late harvest comparison. The number of DEGs in each analysis was compared in a Venn diagram (Figure 1B), where a total of 112 common DEGs were identified in all comparisons. Unexpectedly, the most contrasting comparison (early vs. late) was the one with the lower amount of DEGs, probably because the selected samples of the ‘O×N’ population segregate for other fruit quality traits in addition to the harvest date.

To select only those genes correlated with the harvest date phenotype, the genes differentially expressed between the early and late harvest individuals (1782 DEGs) were filtered considering the expression value of middle harvest individuals. As shown in Table S2, only genes with differential expression between early and late individuals that also had intermediate expression values in middle harvest individuals were selected, eliminating all of those genes that, in middle harvest individuals, had minimum and maximum expression values. Thus, we reduced the number of candidate genes for network construction from 1782 to 1121 DEGs. The resulting 1121 DEGs were presented in a heatmap (Figure 1C), divided into two clusters: the first with 467 DEGs displayed higher expression in the early harvest samples, and the second with 654 DEGs revealed higher expression in the late harvest samples. To perform gene ontology (GO) analysis of both gene clusters, orthologs of *Arabidopsis thaliana* were used. As shown in Figure 1D, gene enrichment was observed with RNA modification or photosynthesis in the 467 DEGs associated with early harvest. In comparison, in the 654 DEGs of late harvest, the GO terms related to cell growth and cell wall remodeling were enriched, among others.

### 2.3. Network Analysis of Differentially Expressed Genes between Early and Late Harvest Samples

From the list of 1121 DEGs, we extracted all of the transcription factors with DAP-seq information in databases that also had high homology with their orthologs in *Arabidopsis thaliana*. The 15 transcription factors identified were classified by the number of targets associated with each one in the same group of 1121 DEGs and filtered by a *p* < 0.01. This strategy reduced the number of transcription factors to nine, with a high number of targets. To construct a network analysis with the most contrasting transcription factors, we only used six, with a |log_2_FC| > 1.0 between the early and late harvest samples. Three of these six candidate transcription factors for harvest date control were identified with higher expression levels in the early harvest samples: the Prupe.2G316600 described as a homeobox-7 (*HB7*) with a log_2_FC = 1.0, the Prupe.7G194400 described as an ethylene-responsive transcription factor (*ERF017*) with a log_2_FC = 2.2 and the Prupe.2G265000 described as a WRKY DNA-binding protein 70 (*WRKY70*) with a log_2_FC = 2.8. The other three were identified with higher expression levels in the late harvest samples: the Prupe.2G200400 described as an MYB-related transcription factor (*LHY*) with a log_2_FC = −1.7, the Prupe.5G194600 described as a cycling DOF factor 3 (*CDF3*) with a log_2_FC = −1.4, and the Prupe.5G146100 described as a NAC domain-containing protein 83 (*NAC083*) with a log_2_FC = −1.5. The six selected transcription factors cover a total of 780 DEGs targets (69.6% of the total selected candidate genes for harvest date). The remaining 30.4% had either no DAP-seq information or were represented by transcription factors with a low number of targets.

**Figure 2:**
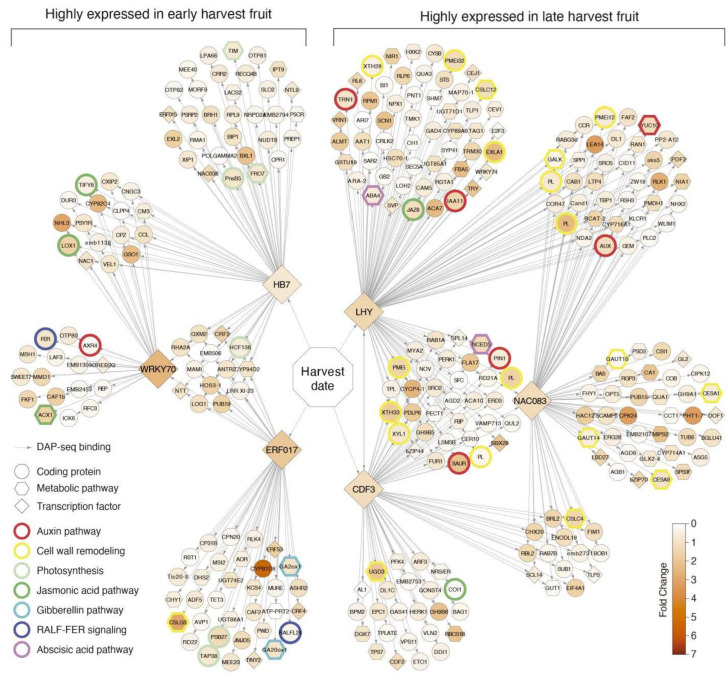
Harvest date network analysis. Representation of most informative genes associated with harvest date regulatory network. Each node represents a differentially expressed gene, and each edge represents a DAP-seq gene association. Orange-scaled colored nodes correspond to the fold change absolute value between early and late harvest comparison. Nodes with colored borders correspond to genes associated with metabolic pathways or signaling.

A network analysis was performed using the six transcription factors described above and the list of their 780 DEGs targets. Figure 2 represents the harvest date regulatory network with the most informative genes. In the group of early harvest overexpressed genes, the presence of genes associated with JA biosynthesis, gibberellic acid (GA) inhibition, RALF-FER signaling (rapid alkalinization factor and feronia signaling), and photosynthesis can be observed (Figure 2, left side). On the other hand, in the late harvest individuals, higher numbers of overexpressed genes associated with auxin biosynthesis, cell wall remodeling and ABA biosynthesis were identified (Figure 2, right side).

### 2.4. Candidate Gene Selection and Evaluation in Contrasting Harvest Date Peach Varieties

Within the 786 DEGs used to build the harvest date regulatory network, 39 candidate genes were selected to explain the harvest date phenotype, including the six candidate transcription factors. These genes are listed in Table 2, with their normalized expression values in early, middle and late harvest samples. The candidate genes were selected considering the enriched functions shown in Figure 2 (auxin pathway, cell wall remodeling, photosynthesis, JA pathway, GA pathway, RALF-FER signaling and ABA pathway) filtered by their expression levels (|log_2_FC| > 0.5 and expression values > 40).

**Figure 3:**
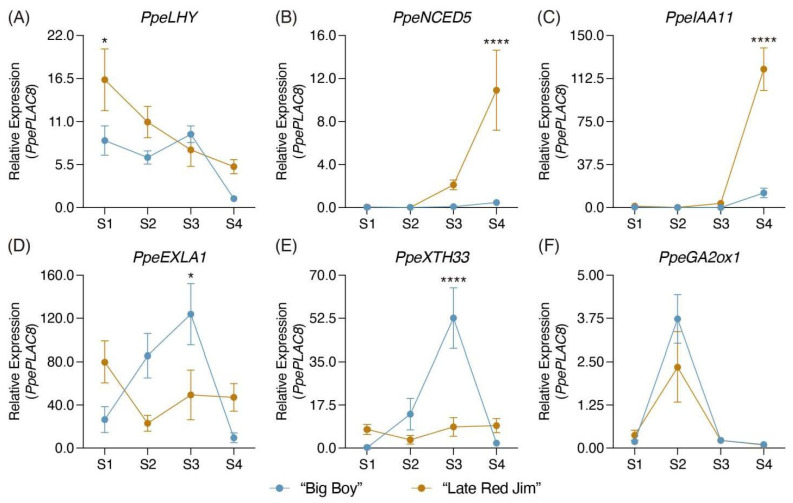
Validation of candidate genes for harvest date phenotype in two contrasting harvest date peach varieties by qPCR. Six candidate genes for harvest date were selected and validated in an early harvest variety “Big Boy” (blue lines) and a late harvest variety “Late Red Jim” (orange lines) in four fruit developmental stages S1, S2, S3 and S4, with S4 being the harvest stage. The Y-axis shows the qPCR relative expression values normalized to the *PpePLAC8* gene. Asterisks show significant differences between “Big Boy” and “Late Red Jim” for each development stage (* *p* < 0.05, **** *p* < 0.0001). (**A**) MYB-related transcription factor LHY (*PpeLHY*; Prupe.2G200400). (**B**) Nine-cis epoxycarotenoid dioxygenase 5 (*PpeNCED5*; Prupe.4G082000). (**C**) Indole-3-acetic acid inducible 11 (*PpeIAA11*; Prupe.7G247500). (**D**) Expansin-like A1 (*PpeEXLA1*; Prupe.8G174500). (**E**) Xyloglucan endotransglucosylase/hydrolase 33 (*PpeXTH33*; Prupe.1G255100). (**F**) Gibberellin 2-β-dioxygenase (*PpeGA2ox1*; Prupe.4G150200).

As a validation of the results obtained for the harvest date phenotypes in the network analysis, six of the network genes were selected and compared in two contrasting peach varieties for harvest date: “Big Boy” and “Late Red Jim”. The expression pattern of the MYB-related transcription factor LHY is shown in Figure 3A (*PpeLHY*). Higher expression levels in the late harvest variety, except in the S3 stage, were observed, as well as a significant differences between both varieties only in stage S1. The two genes selected to validate the participation of ABA and auxins (*PpeNCED5* and *PpeIAA11*, respectively) showed similar behavior (Figure 3B,C, respectively), increasing their expression from the S2 stage and reaching significant differences in stage S4, with higher levels in “Late Red Jim” than “Big Boy” (log_2_FC = 4.5 for *PpeNCED5* and log_2_FC = 3.2 for *PpeIAA11*). Regarding the cell wall remodeling-related genes (*PpeEXLA1* and *PpeXTH33*), it is observed that both genes have higher expression levels in the late harvest variety in stages S1 and S4, while in stages S2 and S3, they present higher expression in the early harvest variety, with statistical differences only in stage S3 (Figure 3D,E). Finally, Figure 3F shows the expression pattern of the *PpeGA2ox1* gene; no significant differences were observed between either variety, but it shows a tendency to present a higher expression level in the “Big Boy” variety in stage S2.

## 3. Discussion

### 3.1. ‘O×N’ Segregating Population Sequencing Evaluation and Bioinformatic Analysis for Harvest Date

A saturated genetic linkage map was constructed for the ‘O×N’ population, and quantitative trait loci (QTL) for harvest date were identified in linkage groups 1, 2, 5 and 6 [30]. A total of 35 candidate genes in these QTL regions with amino acid variations in their protein sequence and probable alterations in their function were selected. Twenty-three of these genes were associated with cell wall remodeling function, among which cellulose synthase, β-glucosidase, polygalacturonase, pectinesterase and pectinesterase inhibitors stood out. In addition, three genes related to JA biosynthesis described as 12-oxophytodienoate reductases (OPR), and eight transcription factors were identified as candidates for harvest date control. Considering these results, RNA samples from early, middle, and late harvest individuals were sequenced. The PCA’s evaluation of the sequenced samples in Figure 1A suggests that the middle harvest samples correspond to the most divergent phenotypic class. The same conclusion is obtained by analyzing the number of DEGs in the three comparisons in Figure 1B. The analysis of early vs late harvest samples had the lowest number of differentially expressed genes (1782 DEGs) compared to the study of early vs. middle or middle vs. late harvest samples (3228 and 3130 DEGs, respectively). The results obtained could be explained by the fact that the selected samples belong to an F1 segregating population that shares an important genetic background and that has low genetic variability. As shown in Table 1, the chosen individuals not only differ in their harvest date, but also segregate for quality traits such as fruit weight, soluble solids content, or even other parameters not evaluated in this study, explaining the low separation of the phenotypic classes observed in the PCA (Figure 1A).

### 3.2. Hormonal Regulation Mechanisms Associated with Late Harvest Samples and Cell Wall Remodeling Enzymes

As shown in Figure 2 (right side), a strong association of the genes related to ABA biosynthesis, cell wall remodeling, auxin biosynthesis, transport and response, and JA inhibition were identified in the late harvest individuals from the ‘O×N’ segregating population.

ABA is a phytohormone synthesized from β-carotene through the action of enzymes such as zeaxanthin epoxidase (ZEP), ABA-deficient 4 (ABA4), 9-cis-epoxycarotenoid dioxygenase (NCED) and ABA-aldehyde oxidase (AAO), among others [31,32]. Several studies showed a high correlation between NCED expression levels and ABA content, an essential gene for this process [33,34]. As displayed in Table 2, a higher expression of key genes in the ABA biosynthesis pathway, *NCED5* and *ABA4*, was associated with the late harvest individuals, probably regulated by the transcription factors *LHY* and *CDF3* (Figure 2), suggesting that there is higher ABA content at harvest in late harvest samples. Similar results were obtained by Hernández et al. [35]. In avocado trees with different physiological ages, they identified a tendency to present a higher hormone content in late harvest fruits, suggesting that ABA has a fundamental role in the harvest date phenotype. Differences in ABA content have been related to changes in many fruit quality traits, such as softening, coloring and biosynthesis of aromatic compounds [18]. ABA exogenous applications have been associated with delays in ripening in the intermediate stages of peach development (S3), correlating to a decrease in the expression of ethylene-, cell wall- and auxin-related genes.

In contrast, when ABA was applicated in stages close to harvest (S4), an inverse effect was observed, promoting the expression of ethylene-, cell wall-, and auxin-related and, in turn, accelerating maturation [36]. Finally, as shown in Figure 3B, the validation of the *NCED5* gene in contrasting varieties for the harvest date phenotype indicates a differential transcript accumulation between “Big Boy” and “Late Red Jim”, suggesting a higher ABA content in the late harvest variety. Considering all of the antecedents described above, we suggest that ABA has a fundamental role in the differences observed between early and late harvest individuals, probably associated with the higher expression of cell wall remodeling and auxin-related genes in late harvest individuals.

As seen in Figure 1D, several GO terms related to the cell wall were associated with the late harvest individuals (cell wall organization and biogenesis, cellulose metabolic and biosynthetic process and cell wall pectin metabolic process). In addition, the network analysis (Figure 2, right side) showed an enrichment of genes with functions associated with cell wall biosynthesis or cell wall remodeling in the late harvest samples, such as xyloglucan endotransglucosylase/hydrolases (*XTH28* and *XTH33*), α-xylosidases (*XYL1*), pectin methylesterase inhibitors (PMEi, PMEi12 and PMEi32), four pectin lyases (PL), expansins (*EXLA1*), cellulose synthases (*CSLC4*, *CSLC12*, *CESA* and *CESA9*), galacturonosyl transferases (*GAUT10* and *GAUT14*) and one UDP-glucose 6-dehydrogenase (*UGD3*), suggesting increased cell wall remodeling activity in late harvest individuals. On the other hand, as mentioned above, previous work carried out in the ‘O×N’ population [30] suggests that genes with cell wall remodeling functions have an important role in harvest date differences. This conclusion was reached by identifying genetic variations that alter the amino acid sequence of genes such as pectin methylesterase inhibitors, cellulose synthases and β-glucosidases. Similar to the results obtained in the network analysis (Figure 2), the validation of the *PpeEXLA1* and *PpeXTH33* genes in the contrasting varieties for harvest date (Figure 3D,E) showed a higher content of both genes in the S4 stage. However, a higher accumulation is also observed in the “Big Boy” variety in earlier stages of fruit development (S2 and S3), suggesting that the cell wall remodeling associated with fruit softening could be out of phase between early and late harvest individuals. All of these antecedents lead us to believe that early and late harvest individuals present differences in fruit cell wall composition or structure. These changes may be related to the differences in ABA- or auxin-related genes. However, more studies are needed to better understand these cell wall changes.

Auxin functions have been associated with plant growth and development, root architecture, phototropism and fruit ripening [37]. In peach fruit, auxins promote the enlargement of mesocarp disc and ripening processes, such as softening and anthocyanin accumulation [38]. As seen in Figure 2, genes related to biosynthesis (*YUC10*), transport (*PIN1* and *TRN1*) and response (*AUX, IAA11* and *SAUR*) of auxins were identified with significantly more expression in the late than in the early harvest samples (Table 2). The same results were obtained from validating the *PpeIAA11* gene in contrasting varieties for the harvest date (Figure 3C), suggesting an increased activity of this hormone in late harvest individuals. Table 1 shows that the fruit weight of late harvest individuals tends to be greater than those of early harvest, a situation also observed in previous studies [10]. Since auxins have a substantial role in growth and development and triggering by an enrichment of genes associated with cell wall remodeling in late harvest individuals, they may be related to or be responsible for this difference in fruit growth. Still, more studies are needed to identify how auxins can control these changes.

### 3.3. Jasmonic Acid Biosynthesis and Gibberellin Inhibition as Hormonal Signals Associated with Early Harvest Samples

In early harvest individuals of the ‘O×N’ segregating population of peach, the enrichment of genes associated with the JA pathway (biosynthesis and repression), gibberellic acid inhibition and photosynthesis-related genes (Figure 2, left side) were obtained.

JA is a fatty acid-derived hormone that can be found conjugated in the form of jasmonate-isoleucine or methyl jasmonate [39]. These endogenous signaling molecules are involved in various developmental processes and were previously known as stress-related hormones in higher plant species [40]. As mentioned above, previous work on the ‘O×N’ population using a genomic approach identified three genes described as 12-oxophytodienoate reductase 2 (*OPR2*) with variations in their amino acid sequence between early and late harvest individuals [30]. OPR genes are part of the JA biosynthesis pathway. Specifically, they reduce the precursor 12-oxophytodienoic acid (OPDA) to synthesize this hormone [41]. In turn, it has been described that OPDA accumulates in the plant and serves as a precursor in JA-independent pathways [42], making the activity of the OPR protein a critical factor in this process. Although further analyses are necessary to determine whether or not these amino acid changes in *OPR2* promote JA biosynthesis in early harvest individuals, it might be possible that JA participates in the regulation of this phenotype in the ‘O×N’ population. In addition, in this work, three genes associated with the JA pathway with higher expression in early harvest individuals were identified, two of them with functions related to its biosynthesis, acyl-CoA oxidase 1 (*ACX1*) and lipoxygenase 1 (*LOX1*), and one related to its repression, TIFY domain protein 8 (*TIFY8*), belonging to the JAZ protein family. While in late harvest individuals, only two genes associated with the repression of this hormone were identified: jasmonate-zim domain protein 8 (*JAZ8*) and coronatine insensitive 1 (*COI1*). These results suggest that there is more ongoing JA biosynthesis in early harvest individuals since only the repression pathway of this hormone is active in late harvest samples.

Contrary to our results, the antecedents of exogenous JA applications in the late stages of fruit development showed that this hormone delays ripening, with fruit displaying higher firmness retention and decreased ethylene biosynthesis [19,22]. It is possible that the changes in JA content between the early and late harvest samples are not only given by their amount in the harvest stage, but could also delay the increase of JA during fruit development. For this reason, measuring JA levels at harvest and at various stages of peach development may be necessary to determine its role in the ripening and harvest date phenotype.

Gibberellic acid (GA) is a tetracyclic di-terpenoid plant hormone that stimulates plant growth and development [43]. The exogenous applications of GA at the end of pit hardening resulted in a delay in peach ripening and increased cell wall material and fruit size, favoring cell expansion and leading to a more significant proportion of cellulose in the cell wall than in the control samples [44,45]. GA is biosynthesized from geranylgeranyl diphosphate, which is transformed into GA_12_ by the activity of the enzymes ent-kaurene synthase (KS), ent-kaurene oxidase (KO) and ent-kaurenoic acid oxidase (KAO). Then, GA_12_ is transformed into bioactive GA by the action of the enzymes gibberellin 20-oxidase (GA20ox) and gibberellin 3-oxidase (GA3ox). However, its activity is regulated by the enzyme gibberellin 2-beta-dioxygenase (GA2ox), which inactivates bioactive GA [46]. We found *GA2ox1* overexpressed at the harvest stage in the early harvest samples, suggesting a lower GA activity in individuals in the late ‘O×N’ population. However, no significant differences in the harvest stage were identified between “Big Boy” and “Late Red Jim” (Figure 3F), so more analyses are necessary to determine the role of GA in the harvest stage between individuals with differences in the harvest date phenotype.

### 3.4. Transcriptional Regulation of Harvest Date Phenotype

The hormonal differences mentioned above in ABA, auxins, JA, GA and even the differences in genes related to cell wall remodeling between individuals with different harvest dates can be observed in Figure 2. Using bioinformatics tools, it was possible to narrow down the regulation mechanisms of these differences to six transcription factors with different expression levels between the early and late harvest individuals. In addition, three transcription factors with higher expression in the early harvest samples (*HB7*, *ERF017* and *WRKY70*) were identified. Likewise, another three transcription factors were detected with higher expression in the late harvest samples (*LHY*, *CDF3* and *NAC083*).

Regarding the transcriptional regulation of early harvest fruit, studies on the transcription factor *HB7* have shown that it responds to water deficit stress and is involved in plant development, delaying the senescence process [47]. It has also been reported to negatively affect ABA signaling through the positive transcriptional regulation of *PP2C* and the transcriptional repression of the ABA receptors *PYL5* and *PYL8* [48]. Although these antecedents agree with the information in Figure 2, possible regulations were only identified between *HB7* with photosynthesis- and JA-related genes (directly or indirectly through the transcription factor *WRKY70*). On the other hand, it has been described that the transcription factor *ERF017* is associated with chlorophyll degradation [49,50]. At the same time, the transcription factor *WRKY70* is considered a modulator between the antagonistic signals of salicylic acid and JA [51]. Considering this background, *WRKY70* seems strongly related to the genes associated with the JA pathway and is likely responsible for the differential expression of JA-related genes between early and late harvest individuals.

Finally, regarding the regulation of differentially expressed genes in the late harvest samples, the role of the transcription factor *CDF3* has been associated with the nitrogen response in tomato [52] and the regulation of flowering time and abiotic stress response induced by drought, extreme salt, temperatures and ABA [53]. At the same time, the transcription factor *NAC083* has been reported as a good indicator of storability in strawberries, correlating the *NAC083* expression pattern with ABA, ethylene and stress-responsive genes [54]. On the other hand, the MYB-related transcription factor (*LHY*) regulates the circadian rhythm in *Arabidopsis thaliana* [55,56]. Previous work reported that *LHY* also bounds the promoter of multiple components of the ABA signaling pathway, promoting the expression of ABA-responsive genes related to increased tolerance to drought and osmotic stress, and decreasing the inhibitory effect of ABA on seed germination and plant growth [57]. In addition, it has been demonstrated that *LHY* and homologous *CCA1* play a pivotal role in gating the auxin response [58]. As displayed in Figure 2, all auxin and ABA candidate genes are targets of *LHY*, in addition to 13 of the 19 cell wall candidate genes of late harvest individuals. On the other hand, *LHY* could regulate the JA balance between early and late harvest individuals because another target is *JAZ8* (a JA signaling inhibitor). Considering that when evaluating the expression pattern of this transcription factor in the “Big Boy” and “Late Red Jim” varieties (Figure 3A), a significant transcript accumulation was identified in the development stages S1, S2 and S4 of the late harvest variety, these results suggest *LHY* as one of the best candidates to explain the observed changes between early and late harvest individuals.

**Figure 4:**
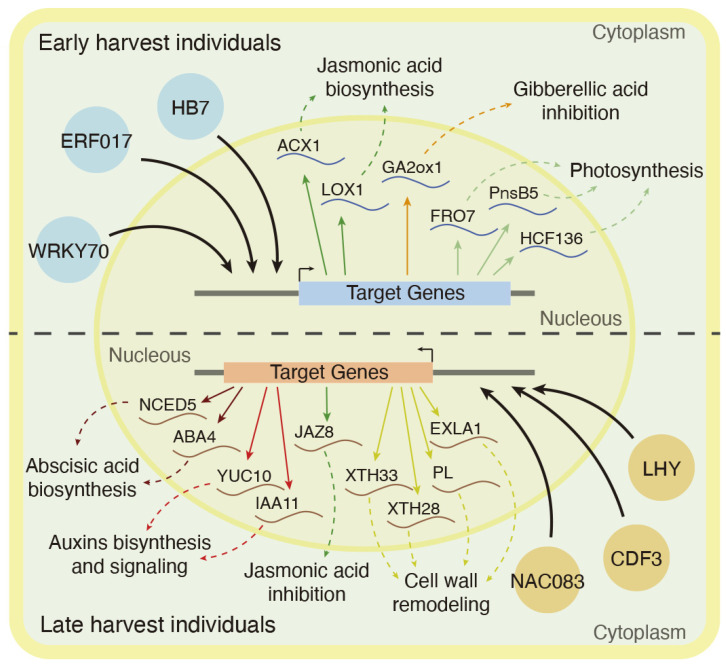
Schematic summary of the significant differences identified between early and late harvest individuals. Early harvest transcriptional regulation is shown on the upper image side, while late harvest transcriptional regulation is shown on the bottom side. Candidate transcription factors are represented in circles, and curve lines represent putative gene targets. Dashed arrows represent hormonal pathways and cell functions associated with each candidate gene.

In conclusion, as shown in Figure 4, genes with higher expression levels in the late harvest samples associated with ABA, auxins and cell wall remodeling were identified (bottom side), while, on the other hand, JA, GA and photosynthesis-related genes were found with higher expression in the early harvest samples (upper side). Finally, through an in silico network analysis, it was determined that the regulation of these changes could be controlled by six transcription factors: *HB7*, *WRKY70* and *ERF017*, with higher expression levels in early harvest individuals, and *LHY*, *CDF3* and *NAC083*, with higher expression levels in late harvest individuals. However, to determine the role in vivo that these transcription factors have in peach fruit quality traits, further analysis, such as yeast one hybrid and/or dual luciferase reporter assays, are necessary.

## 4. Materials and Methods

### 4.1. Plant Material

For the transcriptomic analysis, nine individuals belonging to an F1 population with 194 individuals obtained from the cross between the cultivars “O’Henry” and NR-053 from the Chilean peach breeding program (Universidad de Chile-Andes New Varieties Administration) were evaluated during the 2014–2016 season and selected according to their harvest date in three phenotypic classes (early harvest: O×N-60, O×N-161 and O×N-197; middle harvest: O×N-126, O×N-155 and O×N-172; late harvest: O×N-94, O×N-178 and O×N-180). The harvest date was determined according to the index of absorbance difference (I_AD_), a non-destructively indirect determination of the chlorophyll content in the fruit skin [10]. The I_AD_ value was calculated as the subtraction of absorbance at 670nm minus the absorbance at 720 nm, considering a good I_AD_ value to harvest between 0.8 and 1.2, as described by Lurie et al. [10]. The harvest date is calculated as the number of days after bloom (DAB) until reaching the value of 0.8–1.2 of I_AD_. The “O’Henry” variety produces melting yellow-flesh peach fruit with a late harvest. NR-053 (Maillarmagie cv. Magique^®^) is an early harvest variety that produces melting white-flesh nectarine fruit. The mapping population (‘O×N’) consisted of eight-year-old trees grown on ‘G×N’ rootstock in an experimental orchard in INIA-Rayentué (VI Region, Chile). It was previously used to perform a high-density genetic map and QTL analysis for fruit quality traits such as harvest date, soluble solids content and mealiness [30].

To evaluate the expression levels of the candidate genes, two contrasting varieties for harvest date phenotype (“Big Boy” and “Late Red Jim”) were used to perform RT-qPCR validations. The “Big Boy” and “Late Red Jim” harvest dates were determined according to the parameters used by the orchard in which they are located, considering an adequate firmness to harvest between 13 and 15 N. The I_AD_ values were measured from already harvested fruit. These cultivars produce yellow-flesh nectarines and consist of 7-year-old trees grown on “Nemaguard” rootstocks from the University of Chile Peach Improvement Program (Rinconada, Metropolitan Region, Chile). Each variety was evaluated for fruit quality traits such as fruit size, weight, background fruit color, soluble solids content and harvest date in four developmental stages, considering the average of five fruits during the 2020–2021 season. The fruit growth curve of each variety was analyzed to identify the sampling points corresponding to the development stages S1, S2, S3 and S4 of each variety, with S4 being the harvest date. In this way, the study points evaluated correspond to 43, 64, 85 and 99 DAB for the early harvest variety “Big Boy” and to 39, 79, 149 and 170 DAB for the late harvest variety “Late Red Jim”.

### 4.2. RNA Extraction and Library Construction

Total RNA was extracted from 100 mg of frozen fruit mesocarp of sample points described in the previous section using a Spectrum^TM^ Plant Total RNA kit (Sigma-Aldrich, St. Louis, MO, USA) following the manufacturer’s instructions and stored at −80 °C. RNA quantity was evaluated with a Qubit^®^ 2.0 fluorometer (Invitrogen^TM^, Carlsbad, CA, USA) using a Qubit^TM^ RNA BR assay kit. RNA integrity was assessed by capillary electrophoresis using an Automated CE Fragment Analyzer^TM^ system (Agilent Technologies, Santa Clara, CA, USA) with the RNA kit DNF-471-0500 (15 nt). The RNA quality number (RQN value) was used to identify the integrity of the RNA. RNA samples with an RQN (RNA quality number) value beyond 7.0 were considered with optimal integrity to be used for the following steps.

For the transcriptomic analysis, three selected O×N individuals for each harvest phenotype (early, middle and late harvest) in triplicate at the harvest stage were used for library construction. The RNA libraries were prepared according to the TruSeq Stranded Total RNA Kit (Illumina, San Diego, CA, USA) following the manufacturer’s instructions. The library concentration was determined with a Qubit^®^ 2.0 fluorometer (Invitrogen^TM^) using a Qubit^TM^ dsDNA BR assay kit, and the library size and integrity were evaluated by capillary electrophoresis using the Automated CE Fragment Analyzer^TM^ system (Agilent Technologies) with the DNF-474-0500 HS NGS Fragment Kit. The constructed libraries were sequenced using Macrogen sequencing services (Seoul, Korea) in paired-end mode on a HiSeq4000 sequencer.

### 4.3. Sequencing Data Analysis and Network Construction

Sequencing raw data was evaluated using FASTQC software and filtered with trim-galore v0.6.7 software applying the following criteria: (i) remove adapter sequences; (ii) eliminate reads with a quality score < 25.0; and (iii) eliminate reads with length < 50 nucleotides. The STAR aligner v2.7.10 software [59] was used to align the filtered reads against the *Prunus persica* v2.1 reference genome [29]. For each library, the *featureCounts* function from the Bioconductor-Rsubread package v2.8.1 [60] was applied to assign expression values to each uniquely aligned fragment. Differential gene expression analysis was performed using the Bioconductor-DESeq2 v1.34.0 package, and data normalization was conducted according to the DESeq2 median of ratios method [61]. For a reliable network construction that explains the harvest date phenotype, differentially expressed genes (DEG) were selected only with a false discovery rate of less than 0.05 to use the highest number of harvest date-related genes in the network analysis. The selection of transcription factors was carried out (i) considering the number of target genes of each transcription factor, (ii) a filter of *p* < 0.01 and (iii) that they had the most remarkable differences between early and late harvest individuals |log_2_FC| > 1.0.

Transcriptomic data visualization of all normalized gene counts in a principal component analysis was performed using ggfortify R package v0.4.14 [62] with *autoplot* function, and a heatmap was built to visualize differentially expressed genes using the pheatmap v1.0.12 package. To search for genetic processes and pathways overrepresented in the DEGs lists, a genetic enrichment analysis was performed using the Genetic Ontology (GO) database with the R package ClusterProfiler v4.0.5 [63], using the *compareCluster* function.

A network analysis was conducted using the ConnecTF platform [64], available at https://connectf.org (accessed on 23 November 2022). The list of differentially expressed gene candidates for harvest date was used as “Target Gene List” and “Filter TFs” to identify transcription factor candidates in the ConnecTF database for network construction. The Target List Enrichment tool was used to determine the significance of each transcription factor by comparing the target gene list and queried analyses considering a *p* < 0.01. Finally, network construction was made using Cytoscape software v3.9.1 [65].

### 4.4. RT-qPCR Gene Evaluation in Peach Varieties

The transcript levels of six selected differentially expressed genes were analyzed by qPCR in two contrasting varieties for the harvest date phenotype (“Big Boy” and “Late Red Jim”). For cDNA synthesis, 1 μg of total RNA was first treated with DNase I (Thermo Fischer Scientific, Waltham, MA, USA) and the Superscript II RT system (Invitrogen^TM^) was used according to the manufacturer’s instructions. cDNA synthesis was confirmed by 2.0% agarose gel. RT-qPCR amplification reactions were performed in a total volume of 10.0 μL. The reaction mixture contains 1.0 μL of templated cDNA, 0.5 μL primers (0.25 μL Forward, 0.25 μL Reverse), 0.2 μL ROX, 3.3 μL nuclease-free water and 5.0 μL SYBR Green PCR intercalating dye (Sigma-Aldrich) as a fluorescent indicator. Reactions were performed using three biological and two technical replicates for each evaluation point in the AriaMx Real-Time PCR System (Agilent Technologies). To identify the best housekeeping genes for the qPCR assay, a search for genes with lower differential expression was performed in the RNAseq analysis. Eliminating genes with low levels of expression (<5.0), the *PpePLAC8* gene was selected as housekeeping to calculate the relative expression of our chosen candidate genes. The data were plotted and analyzed in GraphPad Prism v7.05. Statistical analyses were performed using a two-way ANOVA test considering the two peach varieties and the four evaluation points as factors. Significant differences (*p* < 0.05) were expressed by comparing replicate mean values between varieties for each evaluation point.

## Figures and Tables

**Table 1:** Quality traits phenotyping of selected individuals for early, middle and late harvest date belonging to the ‘O×N’ peach population and two harvest date-contrasting peach varieties “Big Boy” (BB) and “Late Red Jim” (LRJ) at harvest stage.

Quality Trait	‘O×N’ Population									Varieties	
	Early Harvest			Middle Harvest			Late Harvest			BB	LRJ
	E60	E161	E197	M126	M155	M172	L94	L178	L180		
I_AD_	1.1 ^a^	1.1 ^a^	1.0 ^a^	1.1 ^a^	1.1 ^a^	1.1 ^a^	1.1 ^a^	1.1 ^a^	0.9 ^a^	0.8 ^A^	1.2 ^A^
Harvest date (DAB)	127.7 ^ab^	123.7 ^a^	132.3 ^ab^	136.0 ^abc^	134.7 ^abc^	139.7 ^bcd^	157.3 ^e^	149.3 ^cde^	153.3 ^de^	104.5 ^A^	170.0 ^B^
Weight (g)	153.6 ^ab^	170.9 ^ab^	154.1 ^ab^	133.3 ^a^	160.7 ^ab^	160.7 ^ab^	164.9 ^ab^	275.2 ^c^	217.5 ^bc^	112.9 ^A^	207.6 ^B^
SSC (ºBrix)	12.4 ^ab^	13.0 ^b^	11.0 ^ab^	12.8 ^b^	12.3 ^ab^	8.8 ^a^	14.4 ^b^	11.2 ^ab^	13.9 ^b^	15.4 ^A^	15.8 ^A^
Firmness (N)	7.0 ^ab^	11.1 ^b^	10.3 ^ab^	10.4 ^ab^	11.2 ^b^	9.1 ^ab^	6.4 ^a^	8.2 ^ab^	6.4 ^a^	14.1 ^A^	15.1 ^A^

Lowercase letters within the row indicate significant statistical differences between individuals of the ‘O×N’ population (*p* < 0.05). Capital letters within the row indicate significant statistical differences between contrasting harvest date peach varieties (*p* < 0.05).

**Table 2:** Candidate genes selected from the transcriptomic analysis for harvest date phenotype in the ‘O×N’ peach population.

PpersicaID	AthalianaID	Symbol	Description	Normalized Expression *		
				Early	Middle	Late
Prupe.2G316600	AT2G46680	HB7	Homeobox 7	172	96	86
Prupe.7G194400	AT1G19210	ERF017	Ethylene-responsive transcription factor ERF017	231	197	50
Prupe.2G265000	AT3G56400	WRKY70	WRKY DNA-binding protein 70	87	18	12
Prupe.2G200400	AT1G01060	LHY	MYB-related transcription factor LHY	122	153	404
Prupe.5G194600	AT3G47500	CDF3	Cycling DOF factor 3	58	103	157
Prupe.1G220400	AT5G13180	NAC083	NAC domain containing protein 83	2234	2459	3404
Prupe.1G478600	AT4G32410	CESA1	Cellulose synthase A1	1308	1814	2255
Prupe.8G035100	AT2G21770	CESA9	Cellulose synthase A9	2104	3247	4602
Prupe.1G418400	AT4G07960	CSLC12	Cellulose-synthase-like C12	596	840	1575
Prupe.3G280100	AT3G28180	CSLC4	Cellulose-synthase-like C4	75	162	241
Prupe.8G174500	AT3G45970	EXLA1	Expansin-like A1	226	511	1407
Prupe.3G258200	AT5G15470	GAUT14	Galacturonosyltransferase 14	793	1003	1339
Prupe.2G206100	AT5G04310	PL	Pectin lyase-like superfamily protein	37	42	137
Prupe.1G129300	AT3G16850	PL	Pectin lyase-like superfamily protein	202	280	393
Prupe.4G271300	AT4G33440	PL	Pectin lyase-like superfamily protein	185	211	264
Prupe.1G114500	AT1G47960	PMEi	Pectin methylesterase inhibitor superfamily	1153	2908	3911
Prupe.7G190400	AT2G26440	PMEi12	Pectin methylesterase inhibitor superfamily	853	1082	1552
Prupe.7G190300	AT3G43270	PMEi32	Pectin methylesterase inhibitor superfamily	1512	3079	4303
Prupe.5G202800	AT5G15490	UGD3	UDP-glucose 6-dehydrogenase family protein	55	113	264
Prupe.1G337000	AT1G14720	XTH28	Xyloglucan endotransglucosylase/hydrolase 28	1819	2545	2868
Prupe.1G255100	AT1G10550	XTH33	Xyloglucan endotransglucosylase/hydrolase 33	327	380	1447
Prupe.1G309900	AT1G68560	XYL1	Alpha-xylosidase 1	439	753	895
Prupe.1G165400	AT3G23805	RALFL24	Ralf-like 24	533	475	360
Prupe.4G150200	AT1G78440	GA2ox1	Gibberellin 2-β-dioxygenase	917	862	391
Prupe.8G192500	AT3G25290	AUX	Auxin-responsive family protein	161	165	412
Prupe.7G247500	AT4G28640	IAA11	Indole-3-acetic acid inducible 11	563	1409	1906
Prupe.5G233100	AT1G73590	PIN1	Auxin efflux carrier family protein	1553	2326	2743
Prupe.4G231800	AT5G55540	TRN1	Tornado 1	108	158	239
Prupe.6G157500	AT1G48910	YUC10	Flavin-containing monooxygenase family	81	179	220
Prupe.5G053500	AT1G67080	ABA4	Abscisic acid (aba)-deficient 4	169	181	302
Prupe.4G082000	AT1G30100	NCED5	Nine-cis-epoxycarotenoid dioxygenase 5	175	2677	5153
Prupe.7G067100	AT4G16760	ACX1	Acyl-CoA oxidase 1	54	18	14
Prupe.4G082500	AT1G30135	JAZ8	Jasmonate-zim-domain protein 8	201	355	515
Prupe.1G467500	AT4G32570	TIFY8	TIFY domain protein 8	68	63	41
Prupe.1G587800	AT5G49740	FRO7	Ferric reduction oxidase 7	75	69	36
Prupe.1G347100	AT5G23120	HCF136	Photosystem II stability/assembly factor	175	134	101
Prupe.1G026900	AT5G43750	PnsB5	NAD(P)H dehydrogenase 18	40	20	19
Prupe.2G313700	AT4G27800	TAP38	Thylakoid-associated phosphatase 38	57	35	29
Prupe.8G160500	AT2G21170	TIM	Triosephosphate isomerase	392	219	197

* Normalized expression values with DESeq2 median of ratios method.

## Data Availability

The datasets generated and analyzed for this study can be found in the National Center for Biotechnology Information (NCBI) repository, PRJNA849174 http://www.ncbi.nlm.nih.gov/bioproject/849174 (accessed on 23 November 2022).

## Supplementary Materials

The following supporting information can be downloaded at: https://www.mdpi.com/article/10.3390/plants11243473/s1, Table S1: Sequencing summary metrics of *Prunus persica* RNA libraries; Table S2: Differentially expressed genes between early and late harvest individuals with normalized expression values and middle harvest expression filtering.
